# Distributions of Direct, Reflected, and Diffuse Irradiance for Ocular UV Exposure at Different Solar Elevation Angles

**DOI:** 10.1371/journal.pone.0166729

**Published:** 2016-11-15

**Authors:** Jiaming Yu, Hui Hua, Yan Liu, Yang Liu

**Affiliations:** 1 Ophthalmology Department, the Fourth Affiliated Hospital of China Medical University, Shenyang, Liaoning, China; 2 School of Public Health, China Medical University, Shenyang, Liaoning, China; 3 Department of Biomedical Engineering, China Medical University, Shenyang, China; Mizoram University, INDIA

## Abstract

To analyze intensities of ocular exposure to direct (E_o,dir_), reflected (E_o,refl_), and diffuse (E_o,diff_) ultraviolet (UV) irradiance at different solar elevation angles (SEAs), a rotating manikin and dual-detector spectrometer were used to monitor the intensity of ocular exposure to UV irradiation (E_o_) and ambient UV radiation (UVR) under clear skies in Sanya, China. E_o,dir_ was derived as the difference between maximum and minimum measured E_o_ values. E_o,refl_ was converted from the value measured at a height of 160 cm. E_o,diff_ was calculated as the minimum measured E_o_ value minus E_o,refl_. Regression curves were fitted to determine distributions of intensities and growth rates at different wavelengths and SEAs. E_o,dir_ differed from ambient UVR exposure. Linear, quadratic, and linear E_o,dir_ distributions were obtained in SEA ranges of 14°–30°, 30°–50°, and 50°–90°, respectively, with maximum E_o,dir_ at 32°–38° SEA. Growth rates of E_o,dir_ with increasing wavelength were fitted with quadratic functions in all SEA ranges. Distributions and growth rate of E_o,refl_ values were fitted with quadratic functions. Maximum E_o,diff_ was achieved at the same SEA for all fitted quadratic functions. Growth rate of E_o,diff_ with increasing wavelength was fitted with a linear function. E_o,dir_ distributions were fitted with linear or quadratic functions in different SEA ranges. All E_o,refl_ and E_o,diff_ distributions were fitted with quadratic functions. As SEA increased, the E_o,dir_ portion of E_o_ increased and then decreased; the E_o,refl_ portion increased from an initial minimum; and the E_o,diff_ portion first decreased and then increased. The findings may provide data supporting on construction of a mathematical model of ocular UV exposure.

## Introduction

Ultraviolet (UV) radiation (UVR) has harmful effects on humans, causing damage to the ocular lens, cornea, and retina [1–3]. Any estimation of the risk of ocular UV damage should include an evaluation of the intensity of ocular exposure to UV irradiation (E_o_). Effects of UVR exposure have been studied in animals, provided important preliminary data [4–6], as well as in human subjects wearing UVR dose detectors [7]. For instance, UVR exposure across the corneal surface was measured in subjects wearing polysulfone contact lenses while walking on a grass field on a cloudy day [8]. In another study, UVR-sensitive films were placed on subjects’ hats, glasses, and chins to evaluate ocular exposure to UV irradiation [9]. Field-based UVR sensors placed on the human body have been used to measure ocular exposure to UV irradiation for a range of solar elevation angles (SEAs), ambient conditions, and head orientations [10]. Manikins have also been used to simulate ocular UV exposure for humans [11, 12].

Ultraviolet (UV) radiation dose has been estimated by using mathematical models, based on data from environmental monitoring, ozone layer thickness, aerosol, cloud thickness, and other parameters. Models have been used to calculate intensities of direct, diffuse, and reflected solar UV irradiance on the horizontal, vertical, and inclined planes [13–22] or in the environment [23, 24]. Researchers have utilized three-dimensional digital models or different body position models to simulate UV exposure [16, 25, 26]. However, because of the complexity of ocular anatomy, monitoring instruments cannot be used to measure directly the intensities of ocular exposure to direct (E_o,dir_), reflected (E_o,refl_) or diffuse (E_o,diff_) UV irradiance.

Previous studies of ocular exposure to UV irradiance have concentrated on monitoring the ocular UV exposure state. To the best of our knowledge, no study has described splitting ocular exposure to UV irradiation into components of direct, reflected, and diffuse UV irradiance. We previously analyzed ocular exposure to UVR at different wavelengths, azimuths, orientations, and reflected backgrounds [10, 11, 27, 28]. We determined that ocular exposure to direct, reflected, and diffuse UV irradiance can be regarded as basic parameters for constructing a mathematical model of ocular UV exposure, which could be used to calculate and compare ocular UV exposure in different regions.

The present study was conducted to monitor the direct, reflected, and diffuse components of ocular exposure to UV irradiation using our self-made ocular UV exposure model. Distributions of ocular exposure to direct, reflected, and diffuse UV irradiance on a rotating manikin were analyzed for different UVR wavelengths and solar elevation angles during the daytime under fine weather in Sanya, China. The goal was to determine the times of maximum ocular exposure to UV irradiation, enabling the prevention of ocular injury from solar UV. Moreover, the findings will provide important data supporting the construction of a mathematical model of ocular UV exposure.

## Material and Methods

### Experimental apparatus

**Fig 1:**
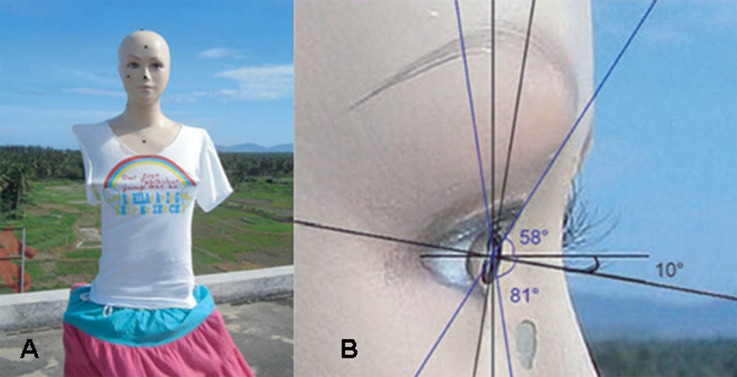
(A) Rotating manikin with solar UV sensor. (B) Details of manikin.

The experimental apparatus was a rotating manikin, which comprised a turntable base, shelf, and anthropomorphic model with realistic facial features (Fig 1). The eye level was at a height of approximately 160 cm. The chosen solar UV sensor, a dual-channel miniature fiber optic spectrometer (AvaSpec-2048x14-2-USB2, Netherlands) with two detectors, was placed on the shelf. One detector was mounted on a plane tangent to the position of the right cornea at the most anterior point on the manikin to record ocular UV exposure (Fig 1A). The other was placed at the vertex of the head of the manikin to record ambient UV irradiance. The visual line was approximately 10° below the horizontal (Fig 1B). The field of view of the manikin was approximately 139°, due to the forehead and malar (Fig 1B). Ground reflection data were obtained with the monitoring instrument at a height of 160 cm and facing the ground. Spectrometer and other equipment were calibrated by the National Physical Laboratory GB before the experiment, as described previously [11].

### Study location

The study site was located in the town of Hai Tang Wan in Sanya city (8.4° N, 109.7° E, altitude 18 m) in the province of Hainan, China. Sanya is the southernmost city in Hainan Island, which has a maximum SEA of nearly 90° in July. The experimental apparatus was located on the asphalt-covered concrete roof of a five-story hotel surrounded by grass with an unobstructed view. The owner of this hotel called ‘‘Dingjun Xu” permitted us to carry out measurements on the roof of his hotel.

### Meteorological conditions

Measurements of UV irradiance exposure were conducted on July 11, 2010 from 08:00 to 19:00 China Standard Time (CST) (solar noon at about 12:55 CST). This day was a sunny day and clear sky.

### UV irradiance measurements

The manikin was rotated clockwise at a constant speed during data collection, beginning with the position of facing the sun. UV irradiance can be monitored (unit μW cm^-2^ nm^-1^) was calculated in 1-s intervals from the integration of the UVA band (320–400 nm) and UVB band (300–320 nm). Duration of each measurement progression was 1 min, and the measurement interval was 5 min. There were 60 groups of irradiance data per manikin revolution. The maximum E_o_ at different wavelengths of each revolution was calculated to simulate the actual maximum UVR exposure under clear skies. The same procedure was used simultaneously to obtain data from the ambient detector.

### Definitions

E_o,dir_ referred to UV irradiation received by the probe placed at the eye position of monitoring model, which consisted of direct solar UV irradiation going straight into the eyes at a certain range of SEAs and/or refraction from direct solar UV hitting the facial structure. E_o,dir_ was measured as the difference between the maximum and minimum values of E_o_ measured from the instrument. Intensity of UV irradiance measured by the equipment at a height of 160 cm was converted to E_o,refl_, according to the structure of the human eye, using the formula [29]:

$$I_{r,\lambda }=0.5S(\lambda )(1-\cos100{}^{\circ})$$

where S(λ) is the measured spectral irradiance. E_o,diff_ was calculated as the difference between the minimum value of measured ocular UV exposure and E_o,refl_.

### Data calculation

Data from the spectrometer were processed with AvaSoft 7.4 USB2.0. Ocular UV exposure and ambient UV data were processed separately. Maximum and minimum integrated E_o_ values of each revolution were calculated from the actual maximum and minimum UV exposures under clear skies. Ocular UV exposure data obtained from the position of the model facing the sun were used to simulate the maximum E_o_ value. When the manikin had its back towards the sun, E_o_ had only reflected and diffuse irradiance dimensions. Data obtained in this position were used as the minimum E_o_ values.

### Monitoring time and SEA

Monitoring time was from 08:00 CST (25° SEA) to 18:00 CST (14° SEA). Range of SEAs was from 14° to 90°, with maximum SEA occurring at about 12:40 CST. According to the relationship between monitoring time and SEA, E_o_ was measured in three ranges of SEA: 14° < SEA ≤ 30° (low), 30° < SEA ≤ 50° (middle) and 50° < SEA ≤ 90° (high).

## Results

### Ambient and ocular UV irradiance at different SEAs

**Fig 2:**
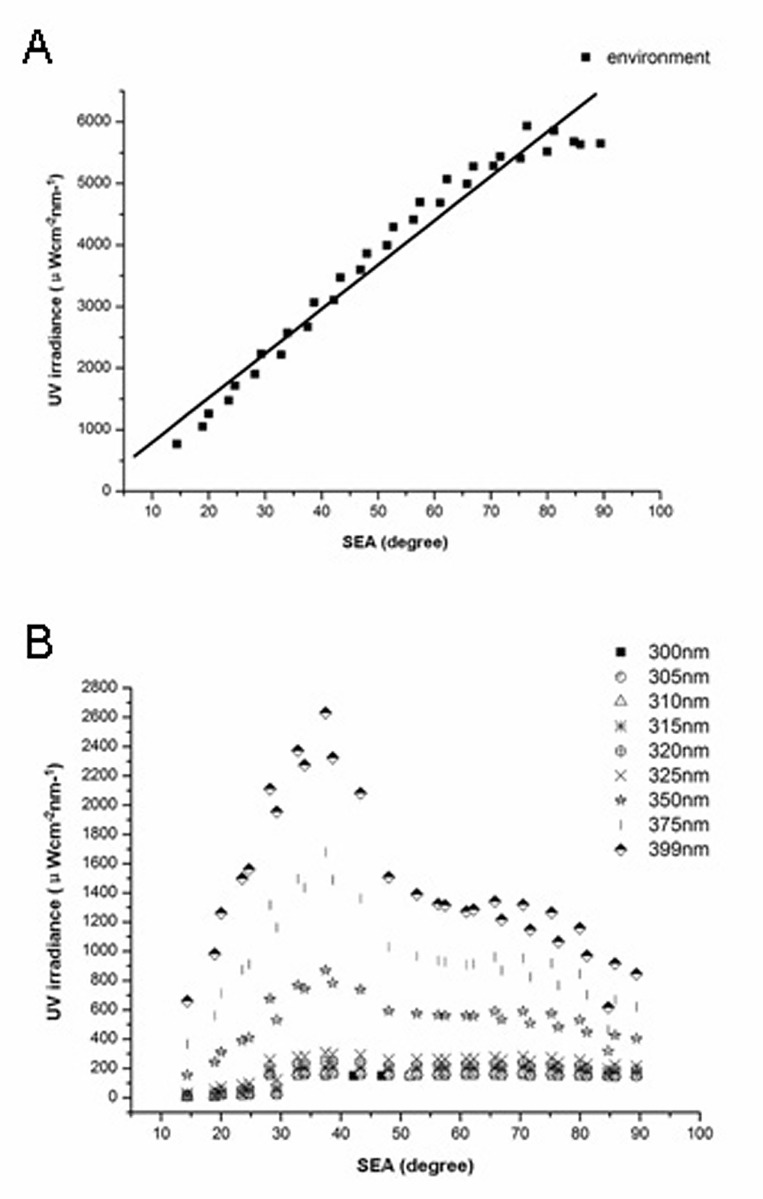
(A) Ambient UV irradiance at different solar elevation angles and fitted regression curve. (B) Ocular UV irradiance of selected wavelengths at different solar elevation angles.

Fig 2 shows the distributions of ambient and ocular UV irradiance intensities at different SEAs. Maximum UV irradiance at each SEA was used as the intensity of ambient UV irradiance (E_amb_). Total E_o_ was determined at nine representative wavelengths of the 300–400 nm UV spectral range, including five wavelengths in the 300–320 nm range (300, 305, 310, 315, and 320 nm) and four wavelengths in the 325–400 nm range (325, 350, 375, and 399 nm).

E_amb_ increased with increasing SEA, with maximum E_amb_ being measured at the highest SEA (Fig 2A). Maximum E_o_ with different wavelengths was achieved in the 30°–40° SEA range (Fig 2B).

### Distributions and growth rates of E_o,dir_ at selected wavelengths and different SEAs

**Fig 3:**
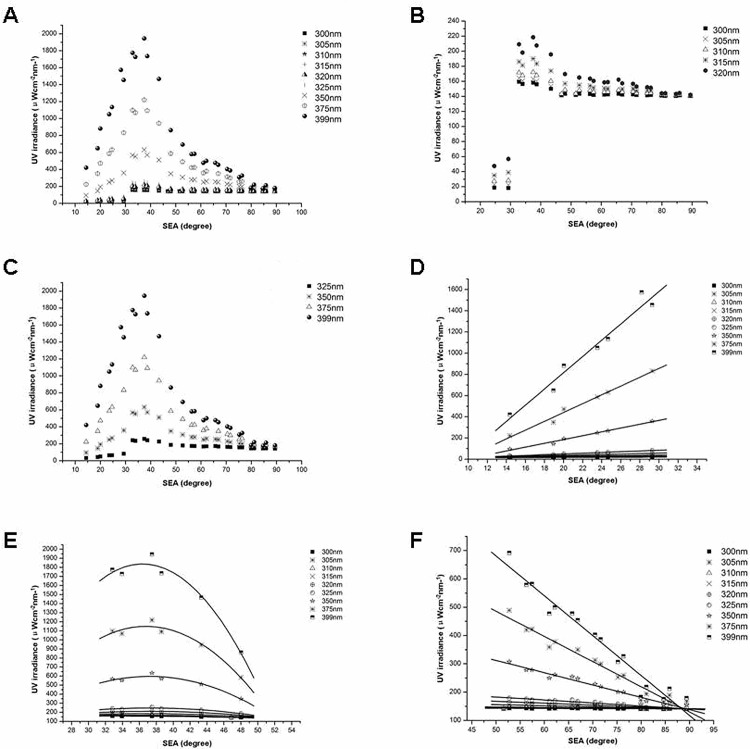
Distributions of intensities and fitted regression curves for ocular exposure to direct UV irradiance of selected wavelengths at different solar elevation angles. (A-C) Intensity distributions at different SEAs for UVR of 300–399 nm (A), 300–320 nm (B), and 325–399 (C). (D-F) Fitted regression curves for all selected wavelengths at low (D), middle (E), and high solar elevation angle range (F).

E_o,dir_ distributions at different SEAs differed markedly from the E_amb_ distribution (Fig 3). In the low range (14°–30° SEA), E_o,dir_ values at different wavelengths increased with increasing SEA, and the distributions were fitted with linear functions (Fig 3D). In the middle range (30°–50° SEA), E_o,dir_ showed binomial distributions, first increasing and then decreasing with SEA, peaking at about 32–38° SEA (Fig 3E). These distributions were fitted with quadratic functions. As wavelength increased in the middle SEA range, the opening of the curve of the quadratic function gradually decreased. In the high range (50°–90° SEA), E_o,dir_ distributions were largely parallel to the x-axis and constant with increasing SEA from 300 to 310 nm, but decreased with increasing SEA from 310 to 400 nm. E_o,dir_ distributions were fitted with linear functions (Fig 3F). For a given SEA, E_o,dir_ increased with increasing wavelength. Equations for regression curves fitted to the E_o,dir_ distributions with increasing SEA are given in Table 1.

**Table 1:** Fitted equations for distributions of ocular exposure to direct UV irradiance of selected wavelengths.

Wavelength	14°-30° SEA		30°- 50° SEA		50°- 90° SEA	
	Fitted equation	R^2^	Fitted equation	R^2^	Fitted equation	R^2^
**305nm**	**y = 0.393x+12.330**	**R**^**2**^ **= 0.787**	**y = -0.095x2+6.337x+57.964**	**R**^**2**^ **= 0.943**	**y = 142.869**	
**310nm**	**y = 0.595x+12.227**	**R**^**2**^ **= 0.890**	**y = -0.147x2+10.174x-5.611**	**R**^**2**^ **= 0.915**	**y = 144.616**	
**315nm**	**y = 1.104x+7.689**	**R**^**2**^ **= 0.980**	**y = -0.211x2+15.219x-88.641**	**R**^**2**^ **= 0.937**	**y = -0.376x+174.630**	**R**^**2**^ **= 0.929**
**320nm**	**y = 2.034x-2.335**	**R**^**2**^ **= 0.998**	**y = -0.361x2+26.845x-288.670**	**R**^**2**^ **= 0.878**	**y = -0.672x+201.360**	**R**^**2**^ **= 0.926**
**325nm**	**y = 3.408x-17.374**	**R**^**2**^ **= 0.995**	**y = -0.526x2+39.129x-479.130**	**R**^**2**^ **= 0.932**	**y = -1.055x+235.670**	**R**^**2**^ **= 0.942**
**350nm**	**y = 17.997x-173.750**	**R**^**2**^ **= 0.986**	**y = -2.135x2+158.770x-2354.500**	**R**^**2**^ **= 0.949**	**y = -4.352x+529.810**	**R**^**2**^ **= 0.958**
**375nm**	**y = 41.533x-389.790**	**R**^**2**^ **= 0.985**	**y = -4.499x2+330.920x-4935.900**	**R**^**2**^ **= 0.961**	**y = -8.887x+930.160**	**R**^**2**^ **= 0.959**
**399nm**	**y = 76.526x-714.560**	**R**^**2**^ **= 0.963**	**y = -7.286x2+530.170x-7806.600**	**R**^**2**^ **= 0.968**	**y = -14.081x+1385.300**	**R**^**2**^ **= 0.957**

**Fig 4:**
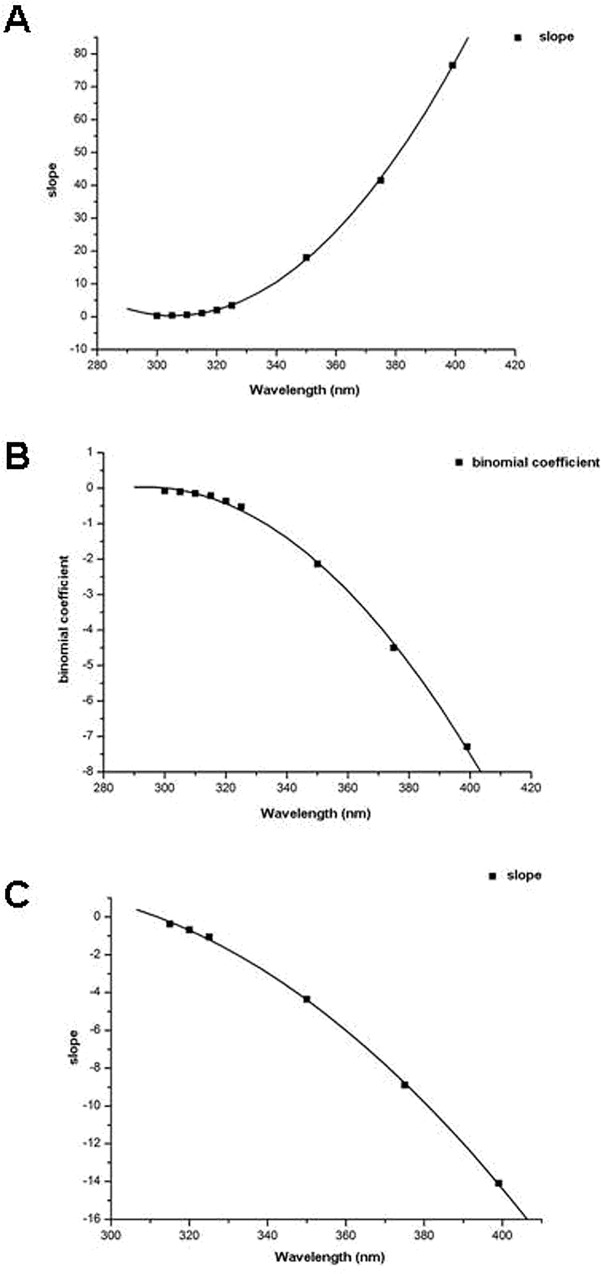
Growth rate of intensity of ocular exposure to direct UV irradiation with increasing wavelength and fitted regression curves in the low (A), middle (B), and high solar elevation angle range (C).

To describe the growth rates of E_o,dir_ values with increasing wavelength, coefficients of each fitted equation in Table 1 were used to fit curves with different wavelengths (Fig 4). In the low SEA range (Fig 4A), the curve slope (gradient) of each linear equation increased with increasing wavelength. The growth rate of curve slopes was fitted with a quadratic function (y = 0.008x2–5.324x + 813.7; R^2^ = 0.999). In the middle SEA range (Fig 4B), binomial coefficients of the fitted equations increased with increasing wavelength. The growth rate of the absolute values of the binomial coefficients was fitted with a quadratic function (y = -0.0007x^2^ + 0.390x - 57.123; R^2^ = 0.999). In the high SEA range (Fig 4C), the curve slope of each linear equation increased with increasing wavelength. The growth rate of the absolute values of the curve slopes was fitted with a quadratic function (y = -0.001x^2^ + 0.5227x - 69.305; R^2^ = 0.9997).

### Distributions and growth rates of E_o,refl_ at selected wavelengths and different SEAs

**Fig 5:**
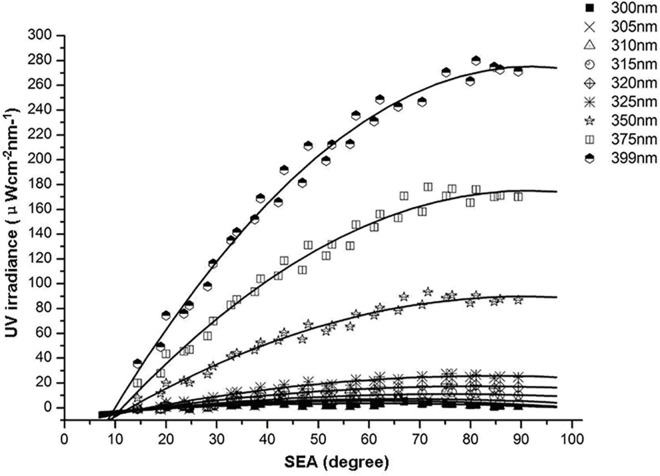
Distributions of intensity of ocular exposure to reflected UV irradiance of selected wavelengths at different solar elevation angles and fitted regression curves.

**Fig 6:**
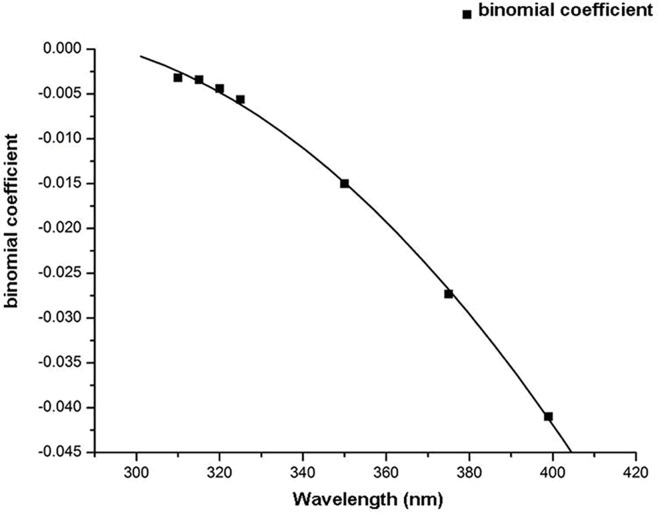
Growth rate of intensity of ocular exposure to reflected UV irradiance with increasing wavelengths and fitted regression curves.

Distributions of E_o,refl_ values at different SEAs differed from the distributions of E_o,dir_ values (Fig 5). Distributions of E_o,refl_ of selected wavelengths increased with increasing SEA and were fitted with quadratic functions (Table 2). The maximum value of each quadratic function increased with increasing SEA or increasing UV wavelength. Absolute values of the binomial coefficients of the fitted equations increased with increasing wavelength (Fig 6). The growth rate of these changes was fitted with a quadratic function (y = -3E-06x^2^ + 0.001x - 0.183; R^2^ = 0.9989). E_o,refl_ increased quickly with increasing SEA or increasing wavelength. The opening of the curve of the quadratic function gradually decreased and the binomial curve become steeper with increasing SEA (Fig 5).

**Table 2:** Fitted equations for distributions of ocular exposure to reflected UV irradiance of selected wavelengths.

Wavelength (nm)	Fitted equation	R^2^
**305**	**y = -0.0029x2+ 0.346x - 5.470**	**R**^**2**^ **= 0.949**
**310**	**y = -0.0032x2+ 0.432x - 7.254**	**R**^**2**^ **= 0.90**
**315**	**y = -0.0034x2+ 0.522x - 8.849**	**R**^**2**^ **= 0.901**
**320**	**y = -0.0044x2+ 0.713x - 11.383**	**R**^**2**^ **= 0.938**
**325**	**y = -0.0056x2+ 0.948x - 14.128**	**R**^**2**^ **= 0.957**
**350**	**y = -0.0150x2+ 2.705x - 32.464**	**R**^**2**^ **= 0.977**
**375**	**y = -0.0273x2+ 4.992x - 53.320**	**R**^**2**^ **= 0.983**
**399**	**y = -0.0410x2+ 7.536x - 70.992**	**R**^**2**^ **= 0.988**

Data represent results for the entire range of solar elevation angles (14–90°).

### Distributions and growth rates of E_o,diff_ at selected wavelengths and different SEAs

**Fig 7:**
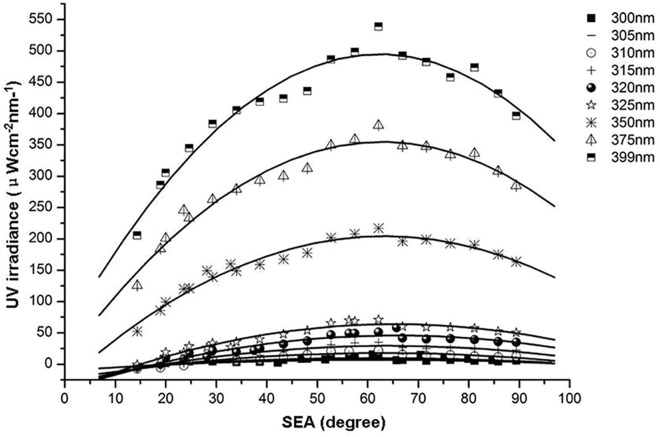
Distributions of intensity of ocular exposure to diffuse UV irradiance of selected wavelengths at different solar elevation angles and fitted regression curves.

**Fig 8:**
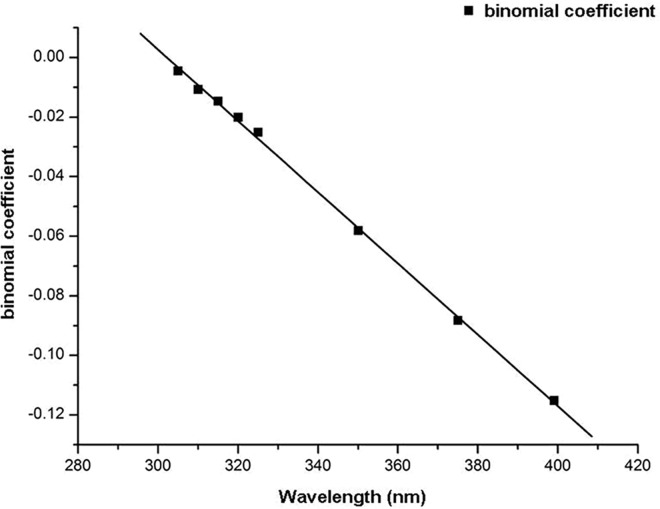
Growth rate of intensity of ocular exposure to diffuse UV irradiance with increasing wavelength and fitted regression curves.

Distributions of E_o,diff_ at different SEAs differed from E_o,dir_ but were similar to E_o,refl_ distributions (Fig 7). At the selected wavelengths, E_o,diff_ first increased and then decreased with increasing SEA. All distributions were fitted with quadratic functions (Table 3). In contrast to E_o,refl_, the maximum E_o,diff_ of each fitted quadratic function with increasing wavelength was achieved at the same SEA of about 62°. Absolute values of the binomial coefficients of each fitted equation increased with increasing wavelength (Fig 8). The growth rate with increasing wavelength was fitted with a linear function (y = -0.0012x + 0.362; R^2^ = 0.999). As wavelength increased, the opening of the curve of the quadratic function decreased at a constant speed. As SEA increased, the rate of change of E_o,diff_ was faster at larger wavelengths (Fig 7).

**Table 3:** Fitted equations for distributions of ocular exposure to diffuse UV irradiance of selected wavelengths.

Wavelength (nm)	Fitted equation	R^2^
**305**	**y = -0.005x2+0.563x-10.429**	**R**^**2**^ **= 0.917**
**310**	**y = -0.011x2+1.346x-24.273**	**R**^**2**^ **= 0.872**
**315**	**y = -0.015x2+1.887x-31.516**	**R**^**2**^ **= 0.915**
**320**	**y = -0.020x2+2.648x-41.236**	**R**^**2**^ **= 0.908**
**325**	**y = -0.025x2+3.288x-43.714**	**R**^**2**^ **= 0.945**
**350**	**y = -0.058x2+7.361x-28.880**	**R**^**2**^ **= 0.962**
**375**	**y = -0.088x2+11.092x+6.200**	**R**^**2**^ **= 0.957**
**399**	**y = -0.115x2+14.370x+46.769**	**R**^**2**^ **= 0.949**

Data represent results for the entire range of solar elevation angles (14–90°).

### Percentages of E_o,dir_, E_o,refl_, and E_o,diff_ in total E_o_

**Fig 9:**
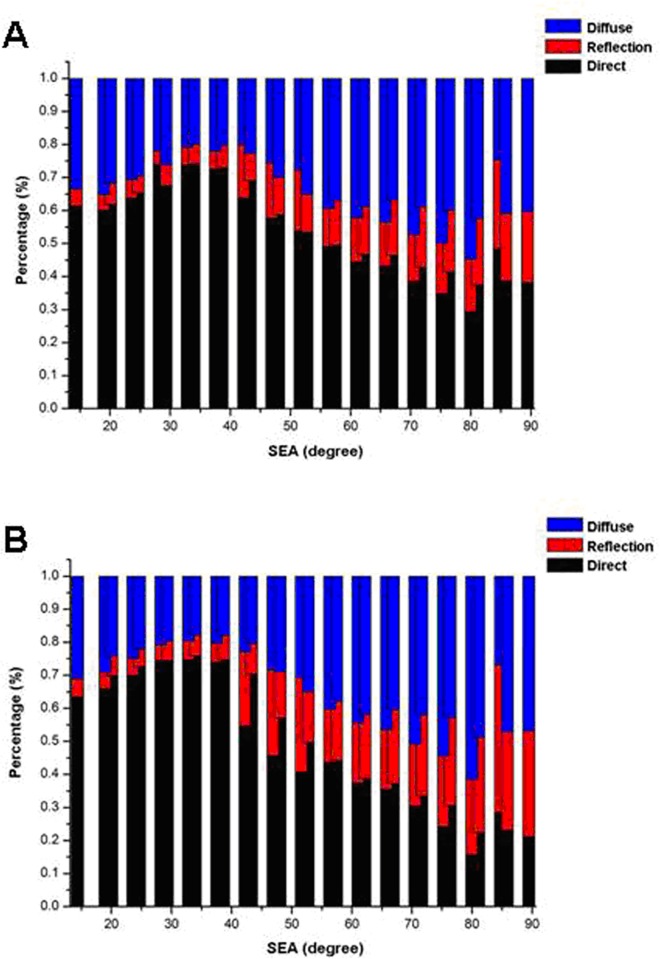
Percentage of total ocular UV exposure due to direct, reflected, and diffuse UV irradiation of 350 nm (A) and 399 nm (B).

Fig 9 shows the percentages of the total E_o_ due to E_o,dir_, E_o,refl_, and E_o,diff_ at 350 nm (Fig 9A) and 399 nm (Fig 9B). As SEA increased, the relative percentages of the three components significantly differed from each other. The percentage due to E_o,dir_ first increased and then decreased with increasing SEA. The percentage due to E_o,refl_ gradually increased from an initial minimum. The percentage due to E_o,diff_ first decreased and then increased.

## Discussion

As we all known, the diurnal distribution of environmental UV exposure is a bell shaped curve, the highest ambient UV irradiances were measured at the highest solar elevation angle, the direction is the dominant in ambient UV irradiances. However, for the ocular UV irradiance, the findings in our previous studies showed the ocular UV irradiances diurnal variations exhibited a bimodal distribution [11, 27, 30], in which the ocular exposure to reflected and diffuse UV irradiance contribute to the bimodal distribution of ocular UV exposure. Therefore, it is necessary to study the distributions of direct, reflected, and diffuse irradiance for ocular UV exposure at different solar elevation angles.

In this study, we found that the distributions of ocular exposure to direct UV irradiance values were different from the distributions of ambient UV irradiance values (which were linear in the entire range of solar elevation angles), but the same as distributions of total ocular exposure to UV irradiation values. Ocular exposure to direct UV irradiance accounted for a large proportion of total ocular exposure to UV irradiation when solar elevation angle was below 50°. In the high solar elevation angle range (50°–90°), ocular exposure to direct UV irradiance decreased with increasing solar elevation angle, but the decreasing trend was significantly faster than that of the total ocular exposure to UV irradiation. One possible reason for this finding is that total ocular exposure to UV irradiation was compensated by the increased impact of ocular exposure to diffuse and reflected UV irradiance.

Distributions of ocular exposure to reflected and diffuse UV irradiance values fit quadratic functions in the entire range of solar elevation angles. The ocular exposure to reflected UV irradiance distribution increased along the entire curve of increasing solar elevation angle, whereas the quadratic curve of ocular exposure to diffuse UV irradiance showed a maximum at about 60° solar elevation angle. The increase of ocular exposure to reflected UV irradiance and the magnitude of changes with increasing solar elevation angle were related to the increased ocular exposure to direct UV irradiance and gradual reduction of the incident angle of reflected light. The ocular exposure to diffuse UV irradiance distribution had a quadratic shape, unlike the largely constant intensity of diffuse UV irradiation. Ocular exposure to diffuse UV irradiance first increased and then decreased with increasing solar elevation angle. One reason for this finding may be that the scattering background causes a deviation of the light scattering angle along with the change of solar elevation angle. Another reason may be that diffusion was affected by changes in ambient UV exposure, which would alter the intensity of diffuse UV irradiation to the eyes.

The percentage of ocular exposure to direct UV irradiance in the total ocular exposure to UV irradiation first increased and then decreased with increasing solar elevation angle. For solar elevation angle below 30°, ocular exposure to direct UV irradiance increased with increasing solar elevation angle, but ocular exposure to reflected and diffuse UV irradiance were less affected. For solar elevation angle above 30°, ocular exposure to reflected and diffuse UV irradiance increased the amount of UV irradiation incoming to the eyes, whereas the proportion of ocular exposure to direct UV irradiance was lower. This result reaffirms the need to prevent excessive ocular exposure to reflected and diffuse UV irradiation in the higher solar elevation angle range.

This study was performed on a clear, fine day in Sanya city. The measurement site has a relatively unpolluted atmosphere (air pollution index < 50 year round), such that the impact of air pollution was negligible. However, the reflection background was an asphalt surface. Distribution characteristics of ocular exposure to UV irradiation components will be different in snow, water, sand, or other backgrounds. In addition, the meaning of “direct” ocular UV exposure was not the same as the physical concept, but was related to our model monitoring conditions. Overall, the results of this study confirm that eye protection should be used at different times throughout the day because of the different proportions of direct, diffuse, and reflected ocular UV irradiation. The findings also support the construction of mathematical models of ocular UV exposure.

## Conclusions

E_o,dir_ distributions were fitted with linear or quadratic functions in different SEA ranges. All E_o,refl_ and E_o,diff_ distributions were fitted with quadratic functions. As SEA increased, the E_o,dir_ portion of E_o_ increased and then decreased; the E_o,refl_ portion increased from an initial minimum; and the E_o,diff_ portion first decreased and then increased.

## Supporting Information

S1 File Monitored data for ocular UV irradiation. (DOC)

S2 File Percentage of direction, reflection and diffusion in total ocular UV irradiation. (DOC)
