# Supplementary material for: Distributions of Direct, Reflected, and Diffuse Irradiance for Ocular UV Exposure at Different Solar Elevation Angles
Source: PLoS One. 2016 Nov 15;11(11):e0166729. doi: 10.1371/journal.pone.0166729 (PMC5112793; doi:10.1371/journal.pone.0166729)
Supplement: S1 File — (DOC) [file pone.0166729.s001.doc]

**Figure 2.** (B) Ocular UV irradiance (unit μW cm^-2^ nm^-1^) of selected wavelengths at different solar elevation angles.

| **Solar Elevation Angle** | **Wavelength (nm)** | | | | | | | | |
| --- | --- | --- | --- | --- | --- | --- | --- | --- | --- |
| **SEA ( ° )** | **300** | **305** | **310** | **315** | **320** | **325** | **350** | **375** | **399** |
| **14.36** | 7.407613 | 9.049763 | 11.02168 | 14.57569 | 21.40513 | 32.52426 | 155.7218 | 367.7676 | 661.5142 |
| **18.94** | 10.96638 | 13.54096 | 16.83018 | 23.27246 | 34.84221 | 52.90906 | 243.6826 | 561.297 | 984.0145 |
| **20.03** | 19.4736 | 23.69056 | 28.69112 | 37.26314 | 51.89155 | 74.15291 | 312.7795 | 716.0301 | 1262.955 |
| **23.55** | 16.57327 | 20.4013 | 26.06675 | 37.42556 | 57.31301 | 87.55151 | 391.2445 | 877.5982 | 1498.706 |
| **24.65** | 21.83943 | 26.50409 | 33.15231 | 45.37695 | 66.25554 | 97.05073 | 409.5519 | 913.1645 | 1562.041 |
| **28.18** | 156.2481 | 160.3856 | 168.0674 | 185.4045 | 215.0984 | 258.3793 | 676.2952 | 1319.253 | 2112.405 |
| **29.30** | 23.54469 | 28.98465 | 37.59615 | 55.00625 | 83.81156 | 125.0666 | 531.5587 | 1165.156 | 1955.64 |
| **32.83** | 156.1398 | 160.9112 | 170.377 | 192.1462 | 228.7426 | 280.8041 | 767.9969 | 1495.675 | 2373.721 |
| **33.96** | 162.0765 | 167.8401 | 178.311 | 199.7822 | 226.4418 | 283.6471 | 744.9544 | 1436.324 | 2274.304 |
| **37.49** | 157.4616 | 163.2156 | 175.8066 | 203.6409 | 248.7451 | 311.207 | 872.5073 | 1680.563 | 2630.424 |
| **38.63** | 162.9216 | 169.1687 | 181.3348 | 206.1416 | 244.8228 | 298.1792 | 783.3957 | 1488.495 | 2324.279 |
| **42.17** | 151.1754 | 154.0407 | 158.8255 | 167.5403 | 180.5084 | 197.6128 | 338.9393 | 527.894 | 738.9429 |
| **43.32** | 160.3445 | 166.4673 | 178.9092 | 203.6173 | 241.2981 | 292.2262 | 738.7248 | 1363.321 | 2082.414 |
| **46.87** | 151.4851 | 154.5324 | 159.9449 | 169.2157 | 182.401 | 199.4274 | 335.0432 | 335.0432 | 699.0347 |
| **48.01** | 155.7942 | 160.8086 | 171.2728 | 191.5951 | 221.7559 | 261.7276 | 593.786 | 1029.798 | 1509.305 |
| **51.57** | 151.3294 | 154.4184 | 159.9302 | 169.6946 | 183.5928 | 201.2022 | 338.6592 | 511.8347 | 699.4745 |
| **52.72** | 156.8869 | 162.2348 | 173.2462 | 194.2487 | 224.518 | 263.6037 | 576.1676 | 970.0159 | 1390.558 |
| **56.28** | 155.9544 | 161.4653 | 172.9202 | 194.3655 | 225.0484 | 264.1345 | 566.3208 | 937.4387 | 1326.703 |
| **57.44** | 157.5113 | 163.2945 | 174.7699 | 195.796 | 225.6771 | 263.8405 | 561.7319 | 929.2496 | 1316.762 |
| **61.00** | 159.5074 | 165.9061 | 178.4 | 200.5543 | 231.1761 | 269.6263 | 560.927 | 911.6531 | 1274.394 |
| **62.16** | 157.6466 | 163.6355 | 175.6075 | 197.2115 | 227.4714 | 265.5238 | 559.0906 | 915.302 | 1286.777 |
| **65.73** | 158.514 | 165.2713 | 178.7104 | 202.8241 | 236.2635 | 278.0728 | 590.6751 | 961.8844 | 1342.804 |
| **66.89** | 154.5087 | 159.7807 | 171.0286 | 191.6937 | 220.4984 | 256.7664 | 534.1589 | 869.0484 | 1218.005 |
| **70.46** | 160.7933 | 168.1802 | 182.3267 | 207.1995 | 241.0342 | 282.855 | 591.7914 | 953.4194 | 1321.345 |
| **71.62** | 151.9132 | 156.8768 | 167.4726 | 187.1337 | 214.5777 | 249.0913 | 511.2205 | 824.7116 | 1149.811 |
| **75.20** | 155.6004 | 162.1426 | 175.6023 | 199.8365 | 233.0793 | 274.2714 | 575.8293 | 920.9254 | 1266.442 |
| **76.36** | 151.7883 | 156.5662 | 166.7967 | 185.4192 | 210.9833 | 242.8931 | 483.6245 | 769.732 | 1067.776 |
| **79.93** | 151.3861 | 156.8012 | 168.7322 | 190.638 | 220.9482 | 258.457 | 533.1938 | 846.6387 | 1159.591 |
| **81.10** | 148.6619 | 152.6696 | 161.6939 | 178.4895 | 201.667 | 230.5261 | 448.7766 | 706.085 | 971.8147 |
| **84.68** | 147.9498 | 150.7989 | 156.5051 | 166.2627 | 179.6191 | 196.1104 | 318.755 | 464.0877 | 616.4722 |
| **85.84** | 147.5058 | 151.1571 | 159.7384 | 175.5374 | 197.3751 | 224.5645 | 428.476 | 668.3198 | 917.2297 |
| **89.41** | 147.7964 | 151.1481 | 158.868 | 173.3703 | 193.4582 | 218.4997 | 404.942 | 622.4855 | 846.9853 |
