# Supplementary material for: Distributions of Direct, Reflected, and Diffuse Irradiance for Ocular UV Exposure at Different Solar Elevation Angles
Source: PLoS One. 2016 Nov 15;11(11):e0166729. doi: 10.1371/journal.pone.0166729 (PMC5112793; doi:10.1371/journal.pone.0166729)
Supplement: S2 File — (DOC) [file pone.0166729.s002.doc]

Table1.Percentage of total ocular UV exposure due to direct, reflected, and diffuse UV irradiation of 350 nm

| **Solar Elevation Angle** | **The percentage in total ocular UV irradiation** | | |
| --- | --- | --- | --- |
| **SEA (º)** | **Direction** | **Reflection** | **Diffusion** |
| **14.36** | 0.61365 | 0.05194 | 0.33442 |
| **18.94** | 0.60141 | 0.04754 | 0.35106 |
| **20.03** | 0.61899 | 0.06390 | 0.31712 |
| **23.55** | 0.63781 | 0.05556 | 0.30663 |
| **24.65** | 0.65434 | 0.04941 | 0.29625 |
| **28.18** | 0.73951 | 0.04001 | 0.22048 |
| **29.30** | 0.67513 | 0.06340 | 0.26146 |
| **32.83** | 0.73827 | 0.05380 | 0.20793 |
| **33.96** | 0.74159 | 0.05902 | 0.19938 |
| **37.49** | 0.72581 | 0.05336 | 0.22083 |
| **38.63** | 0.72987 | 0.06730 | 0.20282 |
| **42.17** | 0.63823 | 0.15972 | 0.20205 |
| **43.32** | 0.69155 | 0.08191 | 0.22654 |
| **46.87** | 0.57773 | 0.16523 | 0.25704 |
| **48.01** | 0.58801 | 0.11328 | 0.29871 |
| **51.57** | 0.53994 | 0.18200 | 0.27806 |
| **52.72** | 0.53485 | 0.11512 | 0.35003 |
| **56.28** | 0.49180 | 0.11569 | 0.39251 |
| **57.44** | 0.49569 | 0.13421 | 0.37010 |
| **61.00** | 0.44586 | 0.13280 | 0.42134 |
| **62.16** | 0.46759 | 0.14400 | 0.38841 |
| **65.73** | 0.43187 | 0.13303 | 0.43510 |
| **66.89** | 0.46528 | 0.16760 | 0.36711 |
| **70.46** | 0.38661 | 0.14037 | 0.47302 |
| **71.62** | 0.42902 | 0.18252 | 0.38846 |
| **75.20** | 0.34855 | 0.15379 | 0.49766 |
| **76.36** | 0.41370 | 0.18739 | 0.39891 |
| **79.93** | 0.29447 | 0.15862 | 0.54690 |
| **81.10** | 0.37471 | 0.20165 | 0.42364 |
| **84.68** | 0.48492 | 0.26829 | 0.24679 |
| **85.84** | 0.38705 | 0.20429 | 0.40865 |
| **89.41** | 0.38285 | 0.21429 | 0.40286 |

Table2.Percentage of total ocular UV exposure due to direct, reflected, and diffuse UV irradiation of 399 nm

| **Solar Elevation Angle** | **The percentage in total ocular UV irradiation** | | |
| --- | --- | --- | --- |
| **SEA (º)** | **Direction** | **Reflection** | **Diffusion** |
| **14.36** | 0.63549 | 0.05388 | 0.31063 |
| **18.94** | 0.65913 | 0.05013 | 0.29074 |
| **20.03** | 0.69905 | 0.05910 | 0.24185 |
| **23.55** | 0.70098 | 0.05070 | 0.24831 |
| **24.65** | 0.72652 | 0.05293 | 0.22055 |
| **28.18** | 0.74511 | 0.04642 | 0.20847 |
| **29.30** | 0.74432 | 0.05951 | 0.19617 |
| **32.83** | 0.74802 | 0.05694 | 0.19504 |
| **33.96** | 0.75960 | 0.06228 | 0.17813 |
| **37.49** | 0.73938 | 0.05784 | 0.20278 |
| **38.63** | 0.74709 | 0.07272 | 0.18018 |
| **42.17** | 0.54737 | 0.22438 | 0.22825 |
| **43.32** | 0.70428 | 0.09204 | 0.20369 |
| **46.87** | 0.45717 | 0.25956 | 0.28327 |
| **48.01** | 0.57102 | 0.13996 | 0.28902 |
| **51.57** | 0.40770 | 0.28480 | 0.30750 |
| **52.72** | 0.49774 | 0.15271 | 0.34956 |
| **56.28** | 0.43663 | 0.16050 | 0.40287 |
| **57.44** | 0.44244 | 0.17905 | 0.37851 |
| **61.00** | 0.37475 | 0.18126 | 0.44399 |
| **62.16** | 0.38833 | 0.19326 | 0.41840 |
| **65.73** | 0.35595 | 0.18085 | 0.46320 |
| **66.89** | 0.37326 | 0.22251 | 0.40423 |
| **70.46** | 0.30524 | 0.18685 | 0.50791 |
| **71.62** | 0.33615 | 0.24442 | 0.41942 |
| **75.20** | 0.24246 | 0.21356 | 0.54398 |
| **76.36** | 0.30620 | 0.26525 | 0.42855 |
| **79.93** | 0.15779 | 0.22720 | 0.61502 |
| **81.10** | 0.22492 | 0.28798 | 0.48711 |
| **84.68** | 0.28548 | 0.44609 | 0.26843 |
| **85.84** | 0.23119 | 0.29756 | 0.47125 |
| **89.41** | 0.21188 | 0.32033 | 0.46778 |
